# Insect Bacterial Symbiont-Mediated Vitellogenin Uptake into Oocytes To Support Egg Development

**DOI:** 10.1128/mBio.01142-20

**Published:** 2020-11-10

**Authors:** Qianzhuo Mao, Wei Wu, Lingzhi Huang, Ge Yi, Dongsheng Jia, Qian Chen, Hongyan Chen, Taiyun Wei

**Affiliations:** a Vector-borne Virus Research Center, Fujian Province Key Laboratory of Plant Virology, Fujian Agriculture and Forestry University, Fuzhou, Fujian, China; b State Key Laboratory for Ecological Pest Control of Fujian and Taiwan Crops, Fujian Agriculture and Forestry University, Fuzhou, China; c College of Life Science, Fujian Agriculture and Forestry University, Fuzhou, China; Johannes Gutenberg University of Mainz; University of Pittsburgh

**Keywords:** bacterial symbionts, embryonic development, leafhopper, Nasuia, vitellogenins

## Abstract

Many insect species, such as aphids, leafhoppers, planthoppers, and whiteflies harbor obligate bacterial symbionts that can be transovarially transmitted to offspring through the oocytes of female insects. Whether obligate bacterial symbionts can carry important molecules/resources to the embryos to support egg development is still unknown. Here, we show that the vitellogenin (Vg) precursor of rice leafhopper Nephotettix cincticeps is biosynthesized by the fat body, secreted into the hemolymph and subsequently cleaved into the 35- and 178-kDa subunits, whereas only the 178-kDa subunit is taken up by the leading end of oocytes in a receptor-dependent manner or moves into the posterior pole of the terminal oocyte in association with obligate bacterial symbiont “*Candidatus* Nasuia deltocephalinicola” (hereafter *Nasuia*) in a receptor-independent manner. Furthermore, the 178-kDa Vg subunit can directly interact with a surface channel molecule (porin) on the envelope of *Nasuia*, allowing Vg to enter bacterial cytoplasm. Thus, Vg can hitchhike the ancient oocyte entry path of *Nasuia*, the common obligate symbiont of leafhoppers. Knocking down a *Nasuia* growth-related protein expression or treatment with porin antibody strongly prevents the ability of *Nasuia* to carry Vgs into oocytes and impair insect egg development. *Nasuia*-carried Vgs provide at least 20% of the total Vgs in the developing eggs. We anticipate that the bacterial symbiont-mediated Vg uptake into oocytes to support efficient egg development may be a common pattern shared by many insects.

## Abstract (precis)

Many insects harbor obligate bacterial symbionts that can be vertically transmitted to offspring by female insects through eggs. Here, we report that leafhopper vitellogenin (Vg) recognizes and binds a surface channel molecule (porin) on the envelope of obligate bacterial symbiont *Nasuia*, which potentially induces the opening of porin channels for Vg to access the cytoplasm of *Nasuia*. Thus, Vg can exploit bacterial symbionts as the independent carriers into the oocytes. Such *Nasuia*-carried Vg contents support efficient insect egg development. Thus, our findings indicate that insects have evolved strategies to exploit the symbionts for carrying additional Vgs to guarantee optimal insect reproduction.

## INTRODUCTION

Many insect species, such as aphids, leafhoppers, planthoppers, and whiteflies, harbor obligate bacterial symbionts, which are often hosted within specialized host cells, namely, bacteriocytes (1, 2). Insect obligate bacterial symbionts are essential for their host’s survival and reproduction, and thus can contribute to host fitness, for example, *Buchnera* spp. of aphids, *Wigglesworthia* spp. of tsetse flies, and *Blochmannia* spp. of ants (2–4). They tend to supplement the host’s diet with amino acids or vitamins that are rare or absent in the food source. Virtually all plant sap-feeding insects have obligate bacterial symbionts providing essential amino acids (4). For example, Buchnera aphidicola synthesizes several amino acids that are required for the aphid’s metabolism (5). When experimentally deprived of the symbiont by antibiotics, host insects suffer retarded growth and high mortality (5). Thus, obligate bacterial symbionts are crucial to improve overall insect host fitness such as providing essential nutrients for survival and reproduction.

Almost all obligate bacterial symbionts in insects are vertically transmitted from a female host to her offspring following their transmission into the developing oocytes at the vitellogenic stage (1, 6, 7). Similarly, the yolk protein precursor vitellogenin (Vg), the essential nutrient for oocyte development in female insects, is also mainly transported into the developing oocytes at the vitellogenic stage (8, 9). Thus, a potential correlation of the oocyte entry for symbionts and Vg might exist. Insect ovaries consist of several ovarioles, each of which contains a germarium, vitellarium, and pedicel. Oocytes produced by the germarium are linearly arranged within the vitellarium and surrounded by a layer of follicular cells (10). Vg is typically biosynthesized extraovarially by the insect fat body, secreted into the hemolymph, taken up specifically by the germarium, and deposited into the oocyte cytoplasm in its storage form, vitellin (9). The Vg protein precursors of most insect species are regulated at the posttranslational level and cleaved into large (140- to 190-kDa) and small (∼50-kDa) subunits before being processed in the ovary (11). It is believed that the large Vg subunits are transported into the rapidly growing insect oocytes through Vg receptor (VgR)-mediated endocytosis (12). Some facultative bacterial symbionts have evolved to hitchhike the existing pathways for Vg to enter insect oocytes. For example, it is thought that *Spiroplasma* in fruit flies (*Drosophila*) or *Wolbachia* in small brown planthoppers utilizes the Vg or yolk entry systems to enter the germarium of the ovary and transport into the oocyte (29). However, whether bacterial symbiont-mediated Vg uptake into the oocytes occurs remains undetermined. We recently reported a new oocyte entry route for viral pathogens by hitchhiking the obligate bacterial symbionts “*Candidatus* Sulcia muelleri” (hereafter *Sulcia*) and “*Candidatus* Nasuia deltocephalinicola” (hereafter *Nasuia*) from the hemolymph into the posterior pole of the terminal oocyte in leafhopper vectors (13, 14). Given that the female insects need to supply the developing eggs with nutrients, it may be possible that the symbionts could also provide a means of transport for host nutrients such as Vg into the eggs.

Leafhoppers (*Cicadellidae*), a large insect family containing approximately 22,000 described species, are cosmopolitan (15). They feed on various crop plants and fruit trees and cause direct damage to agriculture production. Around 200 species of leafhoppers reported can transmit numerous pathogenic microbes (15, 16). Leafhoppers are generally associated with the obligate bacterium *Sulcia* and a betaproteobacterial partner, such as *Nasuia*, which are transmitted via ancient oocyte entry paths from the mother to the offspring (17, 18). *Sulcia* and *Nasuia* provide at least 10 essential amino acids for the survival of host insects (4). In general, *Sulcia* and *Nasuia* leave the bacteriocytes and then simultaneously enter the follicular cells surrounding the posterior poles of the terminal oocytes (epithelial plug) at the vitellogenic stage (17, 18). From there, they directly squeeze and break through the distinct microvilli into the oocytes to form a characteristic “symbiont ball” (17, 18). The rice green leafhopper Nephotettix cincticeps is widely distributed in rice growing countries in Asia. Here, we report that a new oocyte entry route for Vg of N. cincticeps (NcVg) by hitchhiking the obligate bacterial symbiont *Nasuia* from the hemolymph into the posterior pole of the terminal oocyte. Such *Nasuia*-carried NcVgs provide at least 20% of the total Vgs in the developing eggs. Thus, our findings indicate that insects have evolved strategies to exploit the symbionts for carrying additional Vgs to support egg development, possibly improving host fitness.

## RESULTS

### Correlation of the oocyte entry paths for obligate bacterial symbionts and NcVg in *N. cincticeps.*

**FIG 1:**
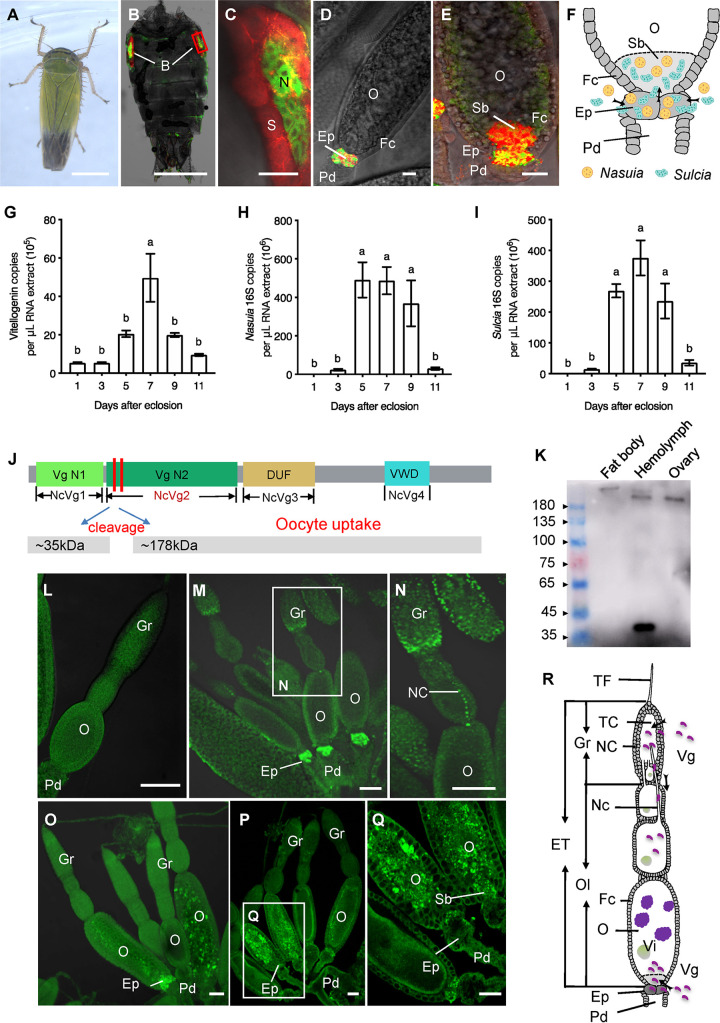
The correlation of the oocyte entry paths for NcVg and the two obligate bacterial symbionts in *N. cincticeps*. (A) A male adult of *N. cincticeps*. Bar, 1 mm. (B and C) *Nasuia* and *Sulcia* inhabit the leafhopper bacteriocytes, as revealed by FISH. Panel C is the enlargement of the red box in panel B. Bars, 1 mm (B) and 50 μm (C). (D and E) *Nasuia* and *Sulcia* inhabit the epithelial plug (D) and entered the posterior pole of the terminal oocyte (E) of the female ovary to form a “symbiont ball” as revealed by FISH. The female abdomens (B and C) and ovaries (D and E) were stained with *Sulcia*-cy5 (red) and *Nasuia*-cy3 (green). Bars, 50 μm. (F) Model for the oocyte entry of *Nasuia* and *Sulcia*. *Nasui*a and *Sulcia* migrated from the hemolymph to the posterior pole of the terminal oocyte via the epithelial plug, and formed a “symbiont ball” within the oocyte cytoplasm. (G to I) The expression patterns of the NcVg gene (G) and the 16S rRNAs of *Nasuia* (H) or *Sulcia* (I) in female ovaries at different developmental stages. Data are presented as means ± standard errors (SE) (error bars) of three independent experiments. The significance of any differences was tested using Tukey’s HSD test. (J) NcVg functional domains and subunit composition, the vitellogenin N1 (NcVg1) and N2 (NcVg2) domains, an unknown function domain (DUF, NcVg3), and a von Willebrand domain (vWD, NcVg4). The cleavage sites were indicated by red lines, and two subunits (35 and 178 kDa) were generated at the cleavage sites. The 178-kDa subunit was sequestered into developing oocytes. (K) NcVg cleavage pattern within the fat body, hemolymph, and ovary of adult female *N. cincticeps* by Western blot analysis. (L to Q) NcVg localization within the ovaries of adult female *N. cincticeps* by immunofluorescence microscopy. The female ovaries at pre-vitellogenic stage (L) and vitellogenic stage (M to Q) were immunolabeled with NcVg-FITC (green). Panels N and Q are enlargements of the boxed areas in panels M and P, respectively. Bars, 100 μm. (R) Proposed model for the oocyte entry of NcVg. During oogenesis, NcVg accumulated in the germarium and then moved to the oocytes via a nutritive cord. NcVg also accumulated in the epithelial plug and moved into the posterior pole of the terminal oocyte to form a “ball.” Abbreviations: B, bacteriocyte; N, nucleus; S, *Sulcia*; ET, egg tube; NC, nurse cell; TF, terminal filament; TC, trophic core; Fc, follicular cell; Gr, germarium; Ep, epithelial plug; Nc, nutritive cord; O, oocyte; Pd, pedicel; Sb, symbiont ball; Vi, vitellin. All images are representative of at least three replicates.

To observe the distribution of the two types of obligate bacterial symbionts *Sulcia* and *Nasuia*, the female adults of *N. cincticeps* were labeled with specific oligonucleotide probes targeting the 16S rRNA of *Sulcia* or *Nasuia* using fluorescence *in situ* hybridization (FISH). We observed that *Sulcia* and *Nasuia* were harbored within the two bacteriocytes in insect abdomens (Fig. 1A to C). FISH and electron microscopy showed that the two symbionts simultaneously entered the epithelial plug (Fig. 1D and F; see also Fig. S1A to G in the supplemental material) and then directly squeezed and broke through the microvilli into the terminal oocyte (Fig. 1E and F and Fig. S1F to I). We found the almost simultaneous appearance of the two symbionts and yolk granules within the oocytes at the vitellogenic stage (Fig. S1J to M), suggesting a potential correlation of the oocyte entry paths for symbionts and NcVg. Furthermore, reverse transcription-quantitative PCR (RT-qPCR) analysis revealed that NcVg transcript levels were significantly increased during the vitellogenic stage and decreased during the post-vitellogenic stage (Fig. 1G). The 16S rRNA levels of *Nasuia* and *Sulcia* in the ovaries were positively correlated with this NcVg accumulation (Fig. 1H and I). These observations indicate that the entry of NcVg into the oocytes may be related to the transovarial transmission of the obligate bacterial symbionts.

10.1128/mBio.01142-20.1 FIG S1 Electron microscopy showed the localization of *Nasuia*, *Sulcia*, and vitellin in the ovaries of female *N. cincticeps* at the vitellogenic stage. (A and B) *Nasuia* and *Sulcia* movement into the epithelial plug from the hemolymph. Panel B is the enlargement of the boxed area in panel A. (C to E) Localization of *Nasuia* and *Sulcia* in the epithelial plug. (F to I) *Nasuia* and *Sulcia* movement from the epithelial plug into the oocyte via squeezing and breaking through the microvilli into the terminal oocyte. (J and K) The simultaneous appearance of the two symbionts and yolk granules within the oocyte cytoplasm. (L and M) The formation of a “symbiont ball” in the oocyte cytoplasm. Panels G, I, K, and M are the enlargements of the boxed areas in panels F, H, J, and L, respectively. Ep, epithelial plug; Sb, symbiont ball; O, oocyte; Vi, vitellin; N, *Nasuia*; S, *Sulcia*; Mv, microvilli. All images are representative of at least three replicates. Bars, 2 μm (A), 500 nm (B), 5 μm (C to K), and 50 μm (L and M). Download FIG S1, TIF file, 2.7 MB. Copyright © 2020 Mao et al. 2020 Mao et al. This content is distributed under the terms of the Creative Commons Attribution 4.0 International license.

To further investigate the correlation of the oocyte entry paths for these two symbionts and NcVg in *N. cincticeps*, we obtained the full-length NcVg cDNA sequence by RT-PCR process (GenBank accession no. KX022097.1). The 6,121-bp NcVg cDNA contained a 6,036-bp open reading frame encoding a deduced 2,011-amino-acid protein (∼220 kDa) and four conserved domains, including two vitellogenin_N domains (NcVg1 and NcVg2), a domain of unknown function (NcVg3), and a von Willebrand factor type D domain (NcVg4) (Fig. 1J and Fig. S2). Furthermore, an amino-terminal sequence analysis demonstrated that the pro-NcVg precursor contained consensus RXXR cleavage site sequences (9) (Fig. 1J and Fig. S2). Vg precursors are generally cleaved into large (140- to 190-kDa) and small (∼50-kDa) subunits before being transported into the ovary (11). The predicted cleavage of NcVg at the consensus RXXR sites in the N-terminal region may generate these large and small subunits (Fig. 1J). Since the NcVg2 domain stretches across the two putative cleaved subunits, we thus prepared NcVg2-specific antibody to investigate the cleavage pattern of pro-NcVg in *N. cincticeps* (Fig. 1J and Fig. S2). We identified intact NcVg (∼220 kDa) in the fat body and two cleaved subunits, 35 and 178 kDa, in the hemolymph, whereas only the 178-kDa subunit was detected in the ovary (Fig. 1K). We then purified the 35- and 178-kDa subunits of NcVg from the hemolymph of the female insects for a mass spectrometry analysis and found that they corresponded to the N and C regions of NcVg, respectively (Fig. S2). These data indicated that pro-NcVg was cleaved at the N-terminal consensus RXXR cleavage site to generate the 35- and 178-kDa subunits in the hemolymph, and only the large 178-kDa NcVg subunit was sequestered by the developing *N. cincticeps* oocytes.

10.1128/mBio.01142-20.2 FIG S2 NcVg cleavage pattern in the leafhopper *N. cincticeps*. Prediction of NcVg domains and cleavage patterns. Amino acid sequences are indicated on colored backgrounds as follows. RXXR cleavage sites are shown on a red background). A polyserine domain is shown on a blue background. Antigens for antibody generation are shown on a gray background. Amino acid sequences of the 35-kDa subunit (blue type) and the 180-kDa subunit (orange type) as revealed by mass spectrometric analysis are shown. Download FIG S2, TIF file, 2.7 MB. Copyright © 2020 Mao et al. 2020 Mao et al. This content is distributed under the terms of the Creative Commons Attribution 4.0 International license.

We then observed the entry dynamics of NcVg in the vitellogenic-stage ovary of the female *N. cincticeps* using immunofluorescence and the NcVg2-specific antibody. NcVg was not visible in the pre-vitellogenic-stage ovary (Fig. 1L); it was initially accumulated in the nurse cells of the germarium and then moved into the oocyte via a nutritive cord in the vitellogenic-stage ovary (Fig. 1M and N) (19). Surprisingly, dense NcVg signals were also observed in the epithelial plug surrounding the posterior pole of the oocyte, where the obligate bacterial symbionts *Sulcia* and *Nasuia* generally accumulated (Fig. 1O). As the oocytes developed, NcVg appeared to form a “ball” and accumulated within the posterior pole of the oocyte (Fig. 1P), before finally moving into the oocytes themselves (Fig. 1P and Q). Collectively, these data indicated that NcVg can be taken up by the germarium and then transported into the oocytes via a nutritive cord and that it may also be transferred directly into the terminal oocytes through the epithelial plug in association with symbiont transmission (Fig. 1R).

### NcVg spreads from the hemolymph into the terminal oocyte in association with *Nasuia.*

**FIG 2:**
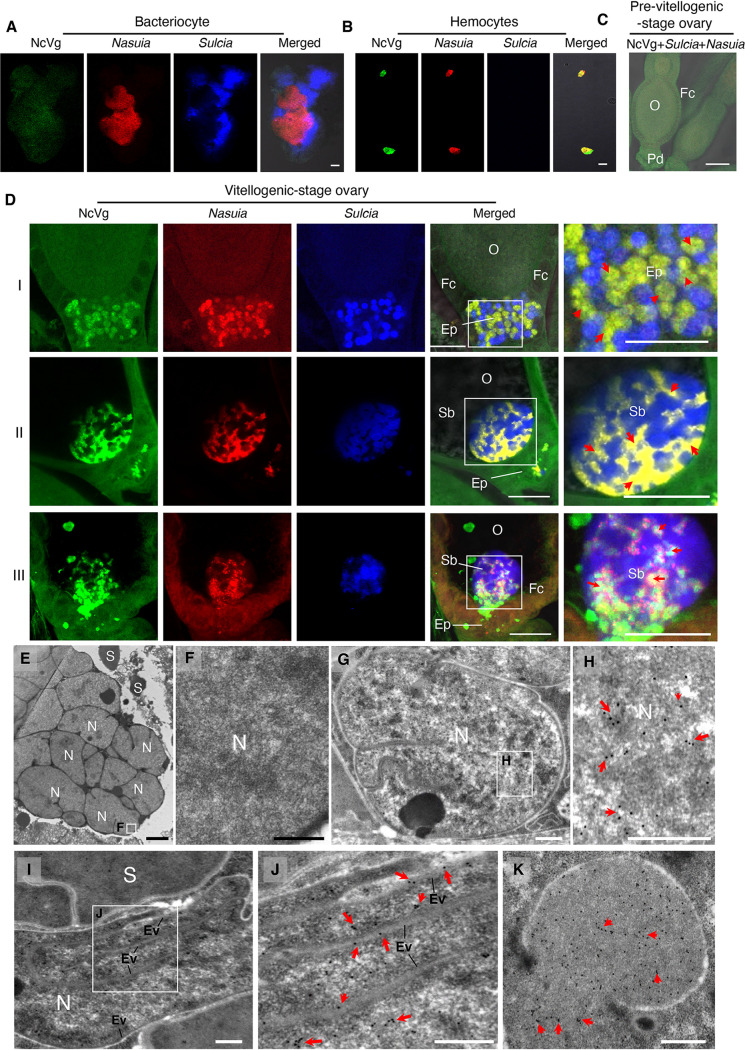
The oocyte entry process of NcVg, *Nasuia*, and *Sulcia* in adult female *N. cincticeps*. (A) Confocal micrographs showed the absence of NcVg within the bacteriocytes containing *Nasuia* and *Sulcia*. Bar, 50 μm. (B) Confocal micrographs showed the colocalization of NcVg with *Nasuia*, rather than with *Sulcia* in the hemocytes. Bar, 25 μm. (C) Confocal micrographs showed that NcVg, *Nasuia*, and *Sulcia* were absent in the epithelial plug of the pre-vitellogenic-stage ovary. Bar, 100 μm. (D) Confocal micrographs showed that NcVg accompanied *Nasuia*, rather than *Sulcia* from the epithelial plug (panels I) into the oocyte to form a “symbiont ball” (panels II), wherein NcVg was released into the oocyte (panels III). Bars, 50 μm. Red arrows marked the colocalization of NcVg and *Nasuia*. The female bacteriocytes (A), hemocytes (B), and ovarioles (C and D) were stained with *Sulcia*-cy5 (blue), *Nasuia*-cy3 (red), and NcVg-FITC (green). (E and F) Immunoelectron micrographs showed that NcVg was absent within the bacteriocytes. Bars, 2 μm (E) and 500 nm (F). (G and H) Immunoelectron micrographs showed the presence of NcVg within the cytoplasm of *Nasuia* in the epithelial plug. Bars, 500 nm. (I and J) Immunoelectron micrographs showed the distribution of NcVg along the envelope invaginations of *Nasuia*. Bars, 500 nm. (K) Immunoelectron micrograph showed the presence of NcVg within the developing yolk granules in the oocyte cytoplasm. Bar, 500 nm. The female bacteriocytes (E and F) and ovaries (G to K) were immunolabeled with NcVg-specific IgG as the primary antibody, followed by treatment with 15-nm gold particle-conjugated goat antibodies against rabbit IgG as the secondary antibody. Panels F, H, and J are the enlargements of boxed areas in panels E, G, and I, respectively. Red arrows mark gold particles. Ep, epithelial plug; Ev, envelope; Fc, follicular cell; Sb, symbiont ball; O, oocyte; N, *Nasuia*; S, *Sulcia*. All images are representative of at least three replicates.

To trace the entry of NcVg, *Nasuia*, and *Sulcia* into the oocytes of *N. cincticeps* adult females, the hemocytes, bacteriocytes, and ovaries were excised and labeled with NcVg2-specific antibody conjugated with fluorescein isothiocyanate (NcVg2-FITC) and specific oligonucleotide probes targeting the 16S rRNA of *Sulcia* or *Nasuia*. Confocal microscopy revealed that NcVg was absent within the bacteriocytes (Fig. 2A) and that it was found to colocalize with *Nasuia* rather than with *Sulcia* in the hemocytes (Fig. 2B and Fig. S3A). Furthermore, NcVg, *Sulcia*, and *Nasuia* were not visible in the pre-vitellogenic-stage ovary (Fig. 2C); however, NcVg accompanied *Nasuia* and was transported into the epithelial plug (Fig. 2D, panels I, and Fig. S3B, panels I) and then into the posterior pole of the terminal oocyte (Fig. 2D, panels II, and Fig. S3B, panels II) in the vitellogenic-stage ovary. Careful observations revealed that NcVg was almost completely colocalized with *Nasuia* signals, suggesting that NcVg was localized inside the cytoplasm of *Nasuia* (Fig. 2D, panels I and II, and Fig. S3B, panels I). Finally, NcVg appeared to be released from the “symbiont ball” into the oocyte cytoplasm (Fig. 2D, panels III, and Fig. S3B, panels III). We examined at least 25 insects with *Nasuia*-associated hemocytes (see Table S1 in the supplemental material) or ovaries (Table S2) and found that NcVg always colocalized with *Nasuia* rather than with *Sulcia*. A parallel immunoelectron microscopy confirmed that NcVg was absent within the cytoplasm of *Nasuia* in the bacteriocytes (Fig. 2E and F) but was present within the cytoplasm of *Nasuia* in the epithelial plug (Fig. 2G and H and Fig. S3C to F). In general, the outer envelopes could form the invaginations within the cytoplasm of *Nasuia*. Immunoelectron microscopy further revealed that NcVg could distribute along the envelope invaginations of *Nasuia* (Fig. 2I and J and Fig. S3G to J). Occasionally, NcVg could be released from the broken envelopes of *Nasuia* (Fig. S3K and L). Thus, we showed the possible pathways for NcVg to release from *Nasuia* by overcoming the bacterial envelope barriers in the terminal oocyte. Finally, the released NcVg can be absorbed by the developing yolk granules in the terminal oocyte (Fig. 2K and Fig. S3M). Among at least 20 insects with *Nasuia*- or *Sulcia*-associated ovariole samples, we calculated that about 80% possessed NcVg-containing *Nasuia*, while none had NcVg-containing *Sulcia* (Table S2). These findings suggest that *Nasuia*-NcVg association may facilitate their simultaneous joint entry into host oocytes, either by hitchhiking the NcVg uptake machinery or being exploited as a transfer vesicle for NcVg.

10.1128/mBio.01142-20.3 FIG S3 The oocyte entry process and distribution of NcVg, *Nasuia*, and *Sulcia* in the ovaries of adult female *N. cincticeps*. (A) NcVg colocalized with *Nasuia*, but not *Sulcia* in the hemocytes. Bar, 10 μm. (B) NcVg accompanied *Nasuia*, rather than *Sulcia* from the epithelial plug (panels I) into the oocyte to form a “symbiont ball” (panels II), and after NcVg was released into the oocyte, NcVg was absent within the “symbiont ball” (panels III). White arrows marked the colocalization of NcVg and *Nasuia*. Bars, 50 μm. The female ovaries were stained with *Sulcia*-cy5 (blue), *Nasuia*-cy3 (red), and NcVg-FITC (green). Pd, pedicel; Fc, follicular cell; Ep, epithelial plug; Sb, symbiont ball; O, oocyte; N, *Nasuia*; S, *Sulcia*. All images are representative of at least three replicates. (C to F) The presence of NcVg within the cytoplasm of *Nasuia* in the epithelial plug. Panels C and E are the enlargements of the boxed areas in panels B and D, respectively. Bars, 500 nm. (G to J) The distribution of NcVg along the envelope invaginations of *Nasuia*. Panels G and I are the enlargements of the boxed areas in panels F and H, respectively. Bars, 500 nm. (K and L) The release of NcVg from the broken envelopes of *Nasuia*. Panel K is the enlargement of the boxed area in panel J. Bars, 500 nm. (M) The presence of NcVg within the developing yolk granules in the oocyte cytoplasm. Bar, 500 nm. The ovaries were immunolabeled with NcVg-specific IgG as the primary antibody, followed by treatment with 15-nm gold particle-conjugated goat antibodies against rabbit IgG as the secondary antibody. Red arrows mark gold particles. Ev, envelope; Vi, vitellin; N, *Nasuia*; S, *Sulcia*. All images are representative of at least three replicates. Download FIG S3, TIF file, 2.8 MB. Copyright © 2020 Mao et al. 2020 Mao et al. This content is distributed under the terms of the Creative Commons Attribution 4.0 International license.

10.1128/mBio.01142-20.7 TABLE S1 Localization of NcVg, *Sulcia.* and *Nasuia* in hemocytes of female *N. cincticeps* as revealed by cofocal microscopy. Download Table S1, DOCX file, 0.02 MB. Copyright © 2020 Mao et al. 2020 Mao et al. This content is distributed under the terms of the Creative Commons Attribution 4.0 International license.

10.1128/mBio.01142-20.8 TABLE S2 Distribution of NcVg in *Sulcia* or *Nasuia* in the ovaries of female *N. cincticeps*. Download Table S2, DOCX file, 0.03 MB. Copyright © 2020 Mao et al. 2020 Mao et al. This content is distributed under the terms of the Creative Commons Attribution 4.0 International license.

### The joint movement of NcVg and *Nasuia* into the oocyte is independent of the NcVgR system.

**FIG 3:**
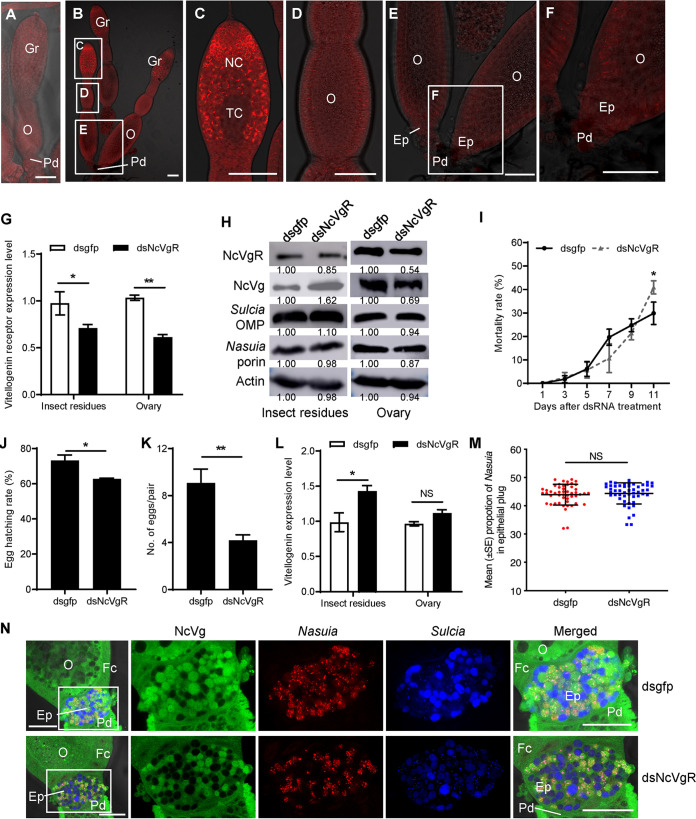
The coentry of *Nasuia* and NcVg into adult female *N. cincticeps* ovary is independent of the NcVgR system. (A to F) Confocal micrographs showed the distribution of NcVgR in the ovaries at the pre-vitellogenic stage (A) and vitellogenic stage (B to F). The ovaries were immunolabeled by NcVgR-rhodamine (red). Panels C, D, and E are enlargements of the boxed areas in panel B. Panel F is an enlargement of the boxed area in panel E. Bars, 100 μm. (G) RT-qPCR assay showed that dsNcVgR treatment significantly decreased the expression levels of NcVgR. (H) Western blot assay showed that dsNcVgR treatment had no significant effect on the abundance of *Sulcia* and *Nasuia* but significantly reduced NcVg accumulation in the ovary and increased NcVg accumulation in other tissues. The protein accumulation levels of NcVgR, NcVg, *Nasuia* porin, and *Sulcia* OMP in insects that received dsgfp were taken to be 1.00. (I) The dsNcVgR treatment showed no statistically significant difference in survival of female *N. cincticeps*. (J to K) The dsNcVgR treatment significantly reduced insect oviposition (J) and egg hatching rate (K) of female *N. cincticeps*. (L) RT-qPCR assay revealed that dsNcVgR treatment significantly increased NcVg expression level in the ovary but had no significant effect on NcVg expression level in other tissues. (M) *Nasuia* movement into the epithelial plug of adult female leafhoppers was not affected after dsNcVgR treatment, as revealed by confocal microscopy. The female ovaries from 50 dsNcVgR- or dsgfp-treated adult female leafhoppers were stained with *Sulcia*-cy5, *Nasuia*-cy3, and NcVg-FITC and observed by confocal microscopy. The number of positive cy3 fluorescent spots in each ovary was calculated to measure the population of *Nasuia*. (N) Confocal microscopy showed the localization of NcVg, *Nasuia*, and *Sulcia* in the epithelial plug of adult female leafhoppers treated with dsNcVgR or dsgfp. The female ovarioles were stained with *Sulcia*-cy5 (blue), *Nasuia*-cy3 (red), and NcVg-FITC (green). Bars, 50 μm. All images are representative of at least three replicates. Data in panels G, I, and J to M are presented as means ± standard deviations (SD) (error bars) of three independent experiments. The significance of any differences was tested using independent *t* test. *, *P* < 0.05; **, *P* < 0.01; NS, not significant.

Given the natural role of VgR as a receptor for Vg, we next asked whether the simultaneous joint entry of *Nasuia* and NcVg into host oocytes is dependent on the VgR-dependent system. We prepared the NcVgR-specific antibody and observed the distribution of NcVgR using immunofluorescence. NcVgR was not visible in the pre-vitellogenic-stage ovary (Fig. 3A); however, it accumulated in the germarium, but not in the epithelial plug surrounding the posterior poles of the terminal oocytes in the vitellogenic-stage ovary (Fig. 3B to F). We thus deduced that the joint movement of NcVg and *Nasuia* into the terminal oocyte is independent of the NcVgR system.

Due to the joint entry of NcVg and *Nasuia* into the terminal oocyte at the vitellogenic stage, we then knocked down the *in vivo* expression of NcVgR (GenBank accession no. KX022098.1) by microinjecting double-stranded RNAs (dsRNAs) targeting this gene (dsNcVgR) into the newly eclosed adult females. Our preliminary experiments showed that microinjection of 3.2 ng dsNcVgR into the bodies of individual female *N. cincticeps* caused an approximately 50% reduction of NcVgR accumulation in the ovaries at the transcript and protein levels (Fig. 3G and H) but only slightly affected leafhopper survival until 11 days (Fig. 3I) and egg hatching rate (Fig. 3J). Although 3.2 ng dsNcVgR treatment delayed the ovary development and reduced fecundity, the ovary still remained intact (Fig. 3K and Fig. S4). In the dsNcVgR-treated females, the accumulation of NcVg decreased by approximately 30% in the ovaries but increased in other tissues at the transcript and protein levels (Fig. 3H and L).

10.1128/mBio.01142-20.4 FIG S4 The female ovary development was delayed by dsNcVgR treatment. (A) Effects of dsgfp or dsNcVgR treatment on ovary development of adult female *N. cincticeps.* Bars, 200 μm. (B) The ovariole of adult female *N. cincticeps* at different developmental stages (I to VI). Bars, 50 μm. (C) Comparison of the number of ovarioles at different developmental stages between dsgfp- and dsNcVgR-treated female *N. cincticeps* at 6 days after microinjection. Thirty insects were collected for each treatment. Download FIG S4, TIF file, 2.6 MB. Copyright © 2020 Mao et al. 2020 Mao et al. This content is distributed under the terms of the Creative Commons Attribution 4.0 International license.

Porin is a class of outer membrane proteins (OMPs) of Gram-negative bacteria (14). Previously, we have prepared antibodies against *Nasuia* porin and *Sulcia* OMP (13, 14). Although the oocyte entry process of *Nasuia* and *Sulcia* was delayed owing to the retarded ovary development following the dsNcVgR treatment (Fig. S4), their abundance remained constant in the ovaries and other tissues, as revealed by Western blot assay with antibodies against *Nasuia* porin and *Sulcia* OMP (Fig. 3H). Confocal microscopy confirmed that *Nasuia*-NcVg colocalization in the ovaries remained congruent after the knockdown of NcVgR expression (Fig. 3M and N). All these results thus provide strong evidence that the entry of the *Nasuia*-NcVg complex into the oocytes is independent of the NcVgR trafficking system.

### *N. cincticeps* exploits *Nasuia* for NcVg transport into the oocytes by establishing a specific interaction between *Nasuia* porin and NcVg.

**FIG 4:**
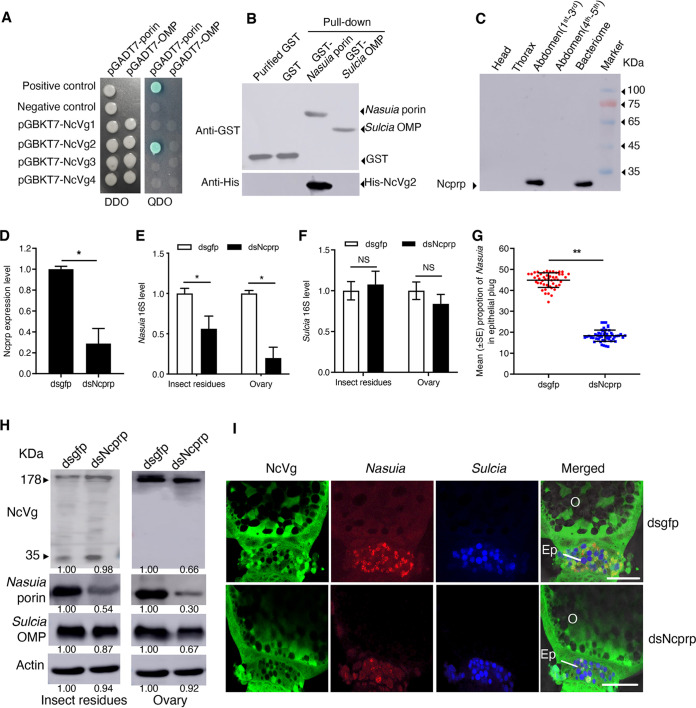
Interaction of NcVg with *Nasuia* porin. (A) Interactions between NcVg1, NcVg2, NcVg3, or NcVg4 with *Nasuia* porin or *Sulcia* OMP in the yeast two-hybrid system. DDO, SD-Trp-Leu; QDO, SD-Trp-Leu-His-Ade. (B) *In vitro* pulldown analysis of interaction of NcVg2 with *Nasuia* porin or *Sulcia* OMP. *Nasuia* porin or *Sulcia* OMP was fused with GST as the bait protein. NcVg2 was fused with His as a prey protein. GST or purified GST proteins were used as controls. (C) Ncprp expression in different tissue parts (head, thorax, abdomen, or bacteriome) of *N. cincticeps* as revealed by Western blot analysis. (D to F) RT-qPCR assay showed that dsNcprp treatment significantly decreased the expression level of Ncprp (D) and reduced the abundance of *Nasuia* (E) but had no significant effect on the abundance of *Sulcia* in *N. cincticep* (F). (G) The reduction of *Nasuia* movement into the epithelial plug of adult female leafhoppers after dsNcprp treatment. The female ovaries from 50 dsNcprp- or dsgfp-treated adult female leafhoppers were stained with *Sulcia*-cy5, *Nasuia*-cy3, and NcVg-FITC and observed by confocal microscopy. The number of positive cy3 fluorescent spots in each ovary was calculated to measure the population of *Nasuia*. (H) NcVg and *Nasuia* porin protein levels decreased but *Sulcia* OMP levels remained constant in dsNcprp-treated female leafhoppers as revealed by Western blot assay. The protein accumulation levels of NcVg, *Nasuia* porin, and *Sulcia* OMP in *N. cincticep* that received dsgfp were taken to be 1.00. (I) Confocal microscopy showed the reduced accumulation of *Nasuia* and NcVg but the normal accumulation of *Sulcia* in the epithelial plug of the oocyte in dsNcprp-treated female leafhoppers. The female ovarioles were stained with *Sulcia*-cy5 (blue), *Nasuia*-cy3 (red), and NcVg-FITC (green). Bars, 50 μm. All images are representative of at least three replicates. Data in panels D to G are presented as means ± SD of three independent experiments. The significance of any differences was tested using independent *t* test. *, *P* < 0.05; **, *P* < 0.01; NS, not significant.

We then investigated how NcVg can reside in the cytoplasm of *Nasuia*. We reasoned that porins, the abundant OMPs in bacterial membranes that form channels to facilitate the passage of some cellular molecules (20), might mediate the entry of NcVg into the *Nasuia* cytoplasm. Yeast two-hybrid assay showed that the NcVg2 domain of Vg precursor can directly interact with *Nasuia* porin (Fig. 4A). Furthermore, NcVg did not interact with the *Sulcia* OMPs (13, 14) (Fig. 4A). A glutathione *S*-transferase (GST) pulldown assay also confirmed that GST-fused *Nasuia* porin specifically bound to His-fused NcVg2 (Fig. 4B). These results suggested that NcVg-porin interaction may activate the porin channel on *Nasuia* envelope to open, allowing NcVg to pass through the envelope into the *Nasuia* cytoplasm.

To further characterize the role of *Nasuia* in the oocyte entry of NcVg, we knocked down the expression of a gene encoding an insect proline-rich protein (N. cincticeps proline-rich protein [NcPRP]) (GenBank accession no. MK722101), which was reported to determine the abundance of *Nasuia* rather than *Sulcia* in *N. cincticeps* by a yet unknown mechanism (21). RT-PCR and Western blot assays revealed an enriched NcPRP expression in the bacteriome but not in other tissues (Fig. 4C and Fig. S5A). Microinjecting synthesized dsRNAs targeting the NcPRP gene (dsNcPRP) into the newly emerged insects resulted in a significant reduction in the abundance of *Nasuia* in both the ovaries and the remaining tissues, while the *Sulcia* abundance remained constant (Fig. 4D to F). The abundance of NcVg in the ovary was decreased in the insects microinjected with dsNcPRP (Fig. 4H), indicating that knocking down NcPRP expression inhibited the oocyte entry of NcVg. Consistently, confocal microscopy indicated that the dsNcPRP treatment almost completely inhibited the coentry of NcVg and *Nasuia* into the epithelial plug and oocyte but did not significantly affect the efficiency of *Sulcia* oocyte entry (Fig. 4H and I). Nevertheless, the dsNcPRP treatment of *N. cincticeps* reduced the survival rate and fecundity of the females, indicating that the *Nasuia* titer was critical for egg maturation (Fig. S5B and C). Together, these results reveal a previously undescribed function for insect obligate bacterial symbionts, wherein *Nasuia* is involved in the process of NcVg oocyte entry.

10.1128/mBio.01142-20.5 FIG S5 Effect of dsNcprp treatment on *N. cincticeps* performance. (A) Detection of Ncprp in different parts of *N. cincticeps* by RT-PCR assay. (B) The mortality rate of *N. cincticeps* increased after dsNcprp treatment. (C) The fecundity of *N. cincticeps* decreased after dsNcprp treatment. Data in panels B and C are presented as means ± SD of three independent experiments. The significance of any differences was tested using independent *t* test. *, *P* < 0.05; **, *P* < 0.01. Download FIG S5, TIF file, 0.3 MB. Copyright © 2020 Mao et al. 2020 Mao et al. This content is distributed under the terms of the Creative Commons Attribution 4.0 International license.

### *Nasuia*-carried NcVg supports efficient egg development.

**FIG 5:**
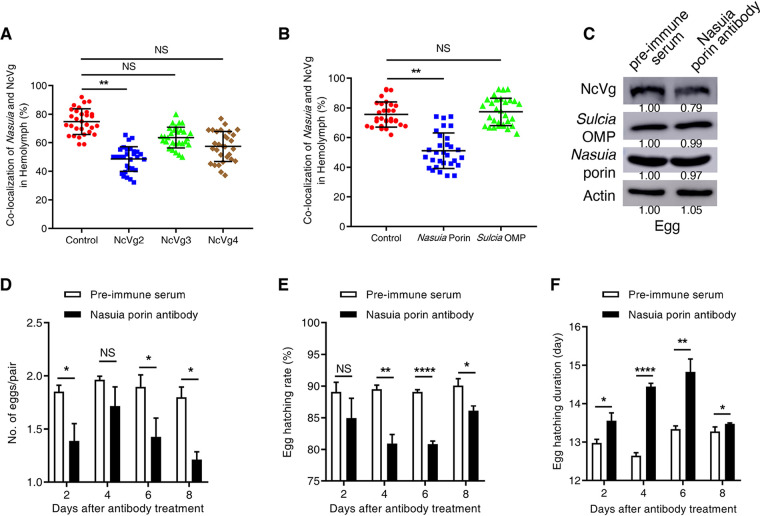
NcVg transport into oocytes is mediated by *Nasuia.* (A) Confocal microscopy showed the reduced colocalization of *Nasuia* with NcVg in the hemolymph of female adults after the treatment with NcVg2 antibody rather than with NcVg3 or NcVg4 antibodies. (B) Confocal microscopy showed the reduced colocalization of *Nasuia* with NcVg in the hemolymph of female adults after the treatment with *Nasuia* porin antibody rather than with *Sulcia* OMP antibody. The hemolymph smears of insects treated with NcVg antibodies (A) or symbiont antibodies (B) were stained with *Nasuia*-cy3 and NcVg-FITC and observed by confocal microscopy. The number of fluorescent spots that were positive for both cy3 and FITC was calculated and represented colocalization of NcVg and *Nasuia*. (C) Western blot assay showed the constant levels of *Nasuia* porin and *Sulcia* OMP in porin antibody-treated eggs laid by antibody-treated adult females but decreased NcVg levels in treated eggs. The protein accumulation levels of NcVg, *Nasuia* porin, and *Sulcia* OMP in the eggs of *N. cincticep* that received preimmune serum were taken to be 1.00. (D) The numbers of eggs laid by adult females treated with porin antibody or preimmune serum were comparable. (E and F) *Nasuia* porin antibody treatment significantly reduced egg hatching rate (E) and prolonged the egg hatching duration (F). Data in panels A, B, and D to F are presented as means ± SD of three independent experiments. The significance of any differences was tested using an independent *t* test. *, *P* < 0.05; **, *P* < 0.01; ****, *P* < 0.0001; NS, not significant.

We next determined how this newly discovered *Nasuia*-mediated oocyte entry pathway for leafhopper Vg influenced the reproduction of the insect hosts. The *in vivo* interaction of NcVg and *Nasuia* porin in the hemocytes was determined by microinjecting antibodies against *Nasuia* porin, *Sulcia* OMP, or NcVg into the insect hemocoel. It was expected that treatment with NcVg2 antibody, but not with NcVg3 or NcVg4 antibodies, caused a significant reduction of the colocalization of *Nasuia* and NcVg in the hemocytes (Fig. 5A). Similarly, the frequency of colocalization of *Nasuia* and NcVg in the hemocytes was significantly decreased after treatment with *Nasuia* porin antibody but remained constant after treatment with *Sulcia* OMP antibody (Fig. 5B). Most importantly, treatment with porin antibody caused an approximately 20% reduction of NcVg deposition in insect eggs without affecting *Sulcia* or *Nasuia* abundance (Fig. 5C). Interestingly, porin antibody treatment significantly reduced insect egg hatching and prolonged the egg hatching period (Fig. 5D to F). Together, these results reveal that *Nasuia*-carried Vgs provided at least 20% of the total Vgs in the developing eggs for efficient embryonic development.

### Interaction of *Nasuia* and Vg in different leafhopper species.

**FIG 6:**
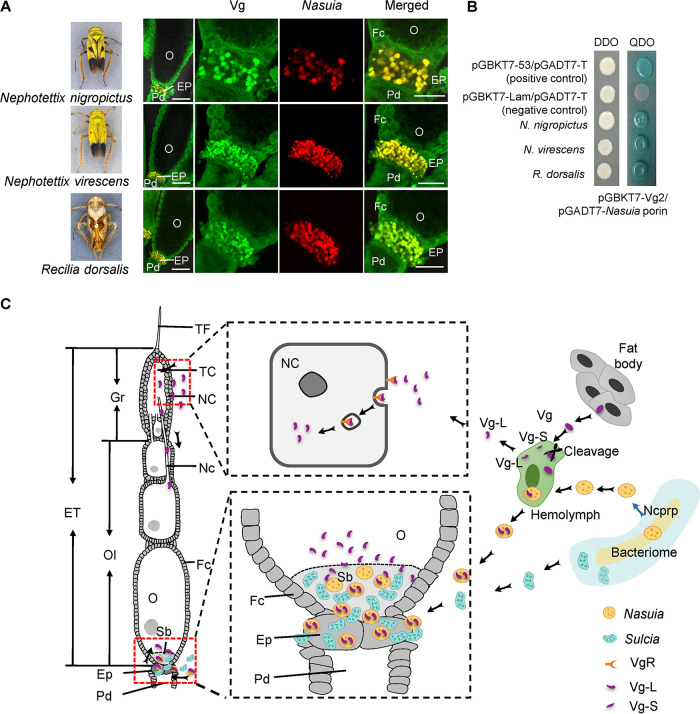
Interactions of Vgs with *Nasuia* porins of different leafhopper species. (A) Localization of Vg and *Nasuia* in the epithelial plugs or the oocytes of the leafhopper *N. nigropictus*, *N. virescens*, or *R. dorsalis*. The female ovaries were stained with *Nasuia*-cy3 (red) and Vg2-FITC (green). Bars in the leftmost insect image panels,  1 mm. Bars in the rightmost immunofluorescence image panels, 50 μm. (B) Interactions between the second domains of Vgs and *Nasuia* porins of the three leafhopper species in the yeast two-hybrid system. DDO, SD-Trp-Leu; QDO, SD-Trp-Leu-His-Ade. (C) Proposed model for the two oocyte entry paths of Vg into insect oocytes. Vgs were synthesized in the fat body and then were processed and cleaved into two subunits, the small subunit (Vg-S, 35 kDa) and the large subunit (Vg-L, 178 kDa) in the hemolymph, and finally, Vg-L was able to enter the developing oocytes via the gemarium entry path or enter the posterior pole of the terminal oocyte via hitchhiking *Nasuia*. ET, egg tube; NC, nurse cell; TF, terminal filament; TC, trophic core; Fc, follicular cell; Gr, germarium; Ep, epithelial plug; Nc, nutritive cord; O, oocyte; Pd, pedicel; Sb, symbiont ball. All images are representative of at least three replicates.

The above results demonstrate that NcVg hitchhikes with the obligate symbiont *Nasuia* for transport into the ovaries of *N. cincticeps*; however, it is unknown whether this phenomenon is unique to this host-symbiont relationship or whether other cases exist. We therefore investigated whether the interaction between *Nasuia* and Vg also occurred in other rice leafhopper species, such as Nephotettix nigropictus, Nephotettix virescens, and Recilia dorsalis (16, 22–24). Phylogenetic analysis of the *Nasuia* 16S rRNA sequences of *N. cincticeps*, N. nigropictus, R. dorsalis, and N. virescens using Bayesian methods revealed that they had formed a close relationship (Fig. S6A). The multiple alignment of the *Nasuia* porin amino acid sequences revealed that the sequences of *N. cincticeps*, *N. nigropictus*, and *N. virescens* were conserved with a high sequence similarity (∼67% identity) but shared a relatively lower similarity with *Nasuia* porin in R. dorsalis (∼41% identity) (Fig. S6B). A sequence alignment of the Vg2 domain in these leafhopper species indicated that they shared high amino acid sequence similarities, ranging from 91% to 95% (Fig. S6C). Furthermore, we examined the localization of *Sulcia*, *Nasuia*, and Vg in the ovaries of these insects, revealing that Vg colocalized with *Nasuia* rather than *Sulcia* in all tested leafhopper species (Fig. 6A). Yeast two-hybrid assay also revealed that *Nasuia* porins of these insect species interacted specifically with their respective Vg2 domains (Fig. 6B). Taken together, these conserved mechanisms indicate that the leafhoppers have evolved to exploit *Nasuia* for transporting Vg into their oocytes by establishing the specific interaction between Vg and the *Nasuia* porin (Fig. 6C).

10.1128/mBio.01142-20.6 FIG S6 Sequence alignment of *Nasuia* porin and Vg2 of different leafhopper species. (A) A phylogenetic tree was constructed based on 16S RNA sequences of *Nasuia* of different leafhopper species by the neighbor-joining method using MEGA7. Numbers at the nodes represent bootstrap values as percentages out of 1,000 replicates. (B) Comparison of amino acid sequences of *Nasuia* porins of *R*. *dorsalis*, *N*. *nigropictus*, *N*. *virescens*, and *N. cincticeps*. The alignment was conducted with DNAMAN software (version 6.0.3.99). (C) Comparison of amino acid sequences of Vg2 of *R. dorsalis*, *N. nigropictus*, *N. virescens*, and *N. cincticeps*. The alignment was conducted with DNAMAN software (version 6.0.3.99). Download FIG S6, TIF file, 2.2 MB. Copyright © 2020 Mao et al. 2020 Mao et al. This content is distributed under the terms of the Creative Commons Attribution 4.0 International license.

## DISCUSSION

To facilitate embryonic development, large quantities of the yolk protein precursor Vgs are transported into insect oocytes through receptor-mediated endocytosis (8, 9). In this study, we report a novel receptor-independent Vg uptake system, in which Vg directly hitchhikes the oocyte entry path of endosymbiont to ensure insect egg development success (Fig. 6C). A rice leafhopper female can oviposit ∼200 eggs at the vitellogenic stage, and thus, massive Vgs are required for egg maturation (25, 26). Rice leafhopper Vg precursor is biosynthesized by the fat body, secreted into the hemolymph for cleavage into the 35- and 178-kDa subunits, whereas only the 178-kDa subunit is taken up by the ovary germarium through the VgR-dependent system (10). Simultaneously, a large amount of *Nasuia* bacteria (5 × 10^8^ copies) leave the bacteriocytes, move into hemolymph, and then move to the posterior poles of the terminal oocytes (14). In the hemolymph, the large cleaved Vg subunits can recognize and bind the porin on *Nasuia* envelope, which potentially induces the opening of porin channels for Vg to pass into the cytoplasm of *Nasuia*. The Vg-containing *Nasuia* then moves into the posterior poles of terminal oocytes. Finally, Vg is potentially released through the porin channels on *Nasuia* envelope invaginations or through the broken *Nasuia* envelopes. The released Vg is ultimately internalized within the developing yolk granules in the oocyte. We calculate that *Nasuia*-carried Vgs provide at least 20% of the total Vg contents in the developing eggs. The supplementation of *Nasuia*-carried Vgs complements the egg development, which guarantees optimal insect reproduction. Notably, the *Nasuia*-mediated oocyte entry of Vg represents a conserved mechanism employed by different leafhopper species, suggesting that this conserved mechanism could play an important role in maintaining insect reproduction.

We anticipate that this newly discovered model for the uptake and storage of Vg by endosymbionts to provide a supplementary source of Vg for egg development may be a common pattern shared by many insects. The posterior pole of the terminal oocyte is the initial oocyte entry site for many obligate bacterial symbionts such as *Buchnera* and *Serratia* in aphids, bacteriocytes in whiteflies, *Sulcia* and *Nasuia* in leafhoppers, and bacteroids in Bradysia tritici (6, 14, 27). However, the leading end of oocytes, the germarium, is the initial oocyte entry site for the facultative bacterial symbionts *Spiroplasma* and *Wolbachia* by hitchhiking the yolk or Vg uptake machinery in a receptor-dependent manner in their respective *Drosophila* or planthopper hosts (28, 29). Here, we find that VgR is absent in the posterior poles of the terminal oocytes of female *N. cincticeps*, from there, *Sulcia* and *Nasuia* directly squeeze and break through the distinct microvilli into the terminal oocyte cytoplasm. Because Vg resides inside the cytoplasm of *Nasuia*, it is reasonable that the transport of Vg-containing *Nasuia* into the terminal oocytes is independent of the Vg/VgR transport and internalization machinery.

Many viral pathogens have evolved to hitchhike the existing pathways for Vg and bacterial symbiont to enter insect oocytes, facilitating the long-term viral epidemic and persistence in nature (2). For example, Vg can carry rice stripe virus or tomato yellow leaf curl virus into the germarium via the VgR-dependent system, from which the virus can spread into the oocyte cytoplasm along the nutritive cords in their respective planthopper or whitefly vectors (19, 30). Our recent finding reveals that rice dwarf virus can recognize and bind *Nasuia* porin to enable virions to pass through the bacterial envelope into the periplasmic space, enabling *Nasuia* to carry virions into the posterior poles of rice leafhopper oocytes (14). We hypothesize that the long-term coexistence of bacterial symbionts, Vg, and viral pathogens on their pathways into insect oocytes may have led to the formation of evolutionary cross-kingdom interactions among them in nature.

Our study demonstrates a novel oocyte entry path for Vg by hitchhiking on *Nasuia* to ensure the success of leafhopper egg development, which guarantees optimal insect reproduction. One female insect generally oviposits dozens or hundreds of eggs at the vitellogenic stage, and thus numerous Vgs are required for egg development and maturation. During insect ovary maturation, the apical and lateral regions of the terminal oocyte are covered by egg envelopes in late vitellogenesis, and consequently, the oocyte entry route for Vg via the nutritive cord may be abolished (7). At this time point, however, the posterior pole of the terminal oocyte is still open for the endosymbionts to enter, and thus, Vg can exploit the terminal oocyte entry paths used by the maternally inherited symbionts. Many insect species, such as aphids, leafhoppers, planthoppers, and whiteflies, harbor obligate bacterial symbionts that are vertically transmitted to the next generation of insects through the posterior poles of the terminal oocytes (1, 2). Given the widespread distribution of these insect species in nature, they may also have gained an evolutionary advantage by hitchhiking with obligate bacterial symbionts for carrying Vg into the posterior poles of the terminal oocytes for ensuing efficient egg development. Specifically, the synchronization of intensive endosymbiont absorption into the ovaries of insect hosts with a massive requirement for Vg during egg maturation minimizes insect investment in Vg uptake and maximizes symbiont transmission.

## MATERIALS AND METHODS

### Measurement of the transcript and protein levels of NcVg, *Nasuia*, and *Sulcia.*

NcVg transcript level and 16S rRNA gene copy number of *Nasuia* and *Sulcia* at 1, 3, 5, 7, 9, and 11 days after emergence were examined. Total RNAs were extracted from 30 adult female leafhoppers, and absolute RT-qPCRs were conducted to analyze NcVg transcript level and 16S rRNA gene copy number. The protein levels of NcVg, *Nasuia* porin, and *Sulcia* OMP in fat bodies, hemolymph, and ovaries of leafhoppers were analyzed by Western blot assay with IgGs specific for NcVg, *Nasuia* porin, and *Sulcia* OMP.

### Immunofluorescence microscopy and fluorescence *in situ* hybridization (FISH).

To examine the oocyte entry process of NcVg, *Nasuia*, and *Sulcia*, 30 ovaries and bacteriocytes from adult female *N. cincticeps* at different days postemergence were dissected, fixed, and immunolabeled with NcVg2 antibody conjugated to FITC (NcVg2-FITC). The ovaries were then fixed, pretreated in hybridization buffer (20 mM Tris-HCl, 180 mM NaCl, 10% sodium dodecyl sulfate, 30% formamide) for 15 min, and incubated in hybridization buffer containing 10 nM oligonucleotide DNA probes, *Sulcia*-cy5 (5′-CTG AAT TAC AAC GTA CAA AAC CC-3′) and *Nasuia*-cy3 (5′-GTA CTA ATT CTT TTA CAA GCA CTT-3′) (Sangon Biotech), which targeted the 16S rRNA sequences of *Sulcia* and *Nasuia*, as described previously with slight modifications (13, 14). After 5-h incubation at 50°C, the samples were thoroughly washed in washing buffer (0.15 M NaCl, 0.015 M sodium citrate) and then observed with a Leica TCS SP5 confocal microscope.

To observe the localization of NcVgR, the ovaries were immunolabeled with NcVgR antibody conjugated to rhodamine (NcVgR-rhodamine) and examined following the above method.

### Electron microscopy.

To observe the subcellular entry process of *Sulcia* and *Nasuia* in ovaries of *N. cincticeps*, 40 ovaries from leafhoppers at different days postemergence were dissected and fixed with 0.5% (vol/vol) glutaraldehyde and 3% (vol/vol) paraformaldehyde in phosphate-buffered saline (PBS) (0.1 M, pH 7.2) for 12 h at 4°C. The fixed tissues were dehydrated in a graded series of ethanol from 50% to 100% at −20°C and embedded in LR Gold resin (SPI Ltd.). For immunoelectron microscopy, ultrathin sections from bacteriocytes and ovaries were immunolabeled with NcVg IgGs as the primary antibody (0.351 mg/ml, diluted with blocking buffer (50 mM PBS [pH 7.0] containing 1% bovine serum albumin [BSA], 0.02% polyethylene glycol 20000 [PEG 20000], and 100 mM NaCl) in 1:200 dilution), followed by treatment with goat anti-rabbit IgG conjugated with 15-nm-diameter gold particles as the secondary antibody (Abcam) (diluted with blocking buffer in 1:100 dilution). The sections were treated only with secondary antibody as a control. Ultrathin sections were examined with an H-7650 Hitachi transmission electron microscope. To determine the distribution relationship of NcVg and the two bacterial symbionts, 23 ovary samples were observed and the distribution was calculated.

### Yeast two-hybrid assay.

To test the interaction between *Nasuia* porin and Vg, a yeast two-hybrid assay was performed using the Matchmaker Gal4 Two-Hybrid System 3 (Clontech). The porin gene of *Nasuia* from *N. cincticeps* was constructed in the prey plasmid pGADT7. Different domains of Vg (Vg1 to Vg4) were constructed in the bait plasmid pGBKT7 (see Table S3 in the supplemental material). The bait and prey plasmids were cotransformed to the yeast strain AH109, and β-galactosidase activity was detected on SD/-Leu/-Trp/-His/-Ade/X-α-Gal culture medium (100 μl of X-α-Gal [4 mg/ml] [catalog no. 630462; Clontech, Mountain View, CA, USA] spread onto a 10-cm SD medium lacking adenine, histidine, leucine, and tryptophan [catalog no. 630323; Clontech, Mountain View, CA, USA], plated using glass beads). The positive-control pGBKT7-53/pGADT7-T and negative-control pGBKT7-Lam/pGADT7-T were transformed in the same way. Since both the gene sequences of *Nasuia* porin and Vg2 from the leafhopper *N. nigropictus*, *N. virescens*, and *R. dorsalis* shared high similarity with those of *N. cincticeps* (Fig. S5B and C), the same primers were used for gene cloning, and the interactions between Vg2 and *Nasuia* porin of the three leafhopper species were monitored in a yeast two-hybrid system.

10.1128/mBio.01142-20.9 TABLE S3 Primers used in this study. Download Table S3, DOCX file, 0.02 MB. Copyright © 2020 Mao et al. 2020 Mao et al. This content is distributed under the terms of the Creative Commons Attribution 4.0 International license.

### GST pulldown assay.

The cDNA fragments of *Nasuia* porin were amplified and cloned into PGEX-3X for fusion with GST. Domain 2 of vitellogenin (NcVg2) was cloned into pDEST17 for fusion with the His tag (Table S3). All recombinant proteins were expressed in Escherichia coli strain Rosetta and purified. GST-*Nasuia* porin was first bound to GST-Sepharose 4B beads (GE) for 3 h at 4°C, then the mixture was centrifuged for 5 min at 100 × *g*, and the supernatant was discarded. Histidine-tagged NcVg2 (His-NcVg2), NcVg3, or NcVg4 was added to the beads and incubated for 2 h at 4°C. After being centrifuged and washed five times with washing buffer (300 mM NaCl, 10 mM Na_2_HPO_3_, 2.7 mM KCl, and 1.7 M KH_2_PO_4_), the bead-bound proteins were separated by sodium dodecyl sulfate-polyacrylamide gel electrophoresis (SDS-PAGE) and detected by Western bloting with His-tagged antibody and GST-tagged antibody (Sigma).

### Neutralizing Vg-*Nasuia* binding *in vivo*.

To examine the binding affinity of NcVg to *Nasuia* porin in hemocytes of *N. cincticeps*, we microinjected the antibodies against *Nasuia* porin, *Sulcia* OMP, or NcVg into insect hemocoel to neutralize the interaction. The abdomens of 30 adult female leafhoppers at 1 day after emergence were microinjected with 64 nl of antibody solution (0.5 μg/μl) or preimmune serum (0.5 μg/μl) as a control, and the leafhoppers were allowed to feed on healthy rice seedlings. The hemolymph collected at 3 days after microinjection was immunolabeled with NcVg-FITC and hybridized with the *Sulcia*-cy5 or *Nasuia*-cy3 probe and observed by confocal microscopy. To calculate the colocalization of NcVg and *Nasuia*-cy3, 30 insect hemolymph samples for each treatment were prepared.

To determine the role of *Nasuia*-mediated Vg transfer manner in insect reproduction, the adult female leafhoppers at 5 days after emergence were microinjected with *Nasuia* porin antibody or preimmune serum and were allowed to feed on rice seedlings for oviposition. At 7 days postoviposition, the number of eggs laid by the female was recorded and the duration and hatching rate were measured. The protein levels of NcVg, *Nasuia* porin, and *Sulcia* OMP in 100 eggs from each treatment group were detected by Western blot assay.

### Knocking down the *in vivo* expression of NcVgR in *N. cincticeps*.

In order to examine whether *Nasuia* hitchhiked NcVg transport system, RNA interference (RNAi) was performed to knock down expression of the NcVgR gene. The abdomens of newly emerged adult females of *N. cincticeps* were microinjected with dsRNAs targeting NcVgR gene (dsNcVgR) or the green fluorescent protein (gfp) gene (dsgfp), and the treated insects were then allowed to feed on healthy rice seedlings. At 6 days after microinjection, 50 ovaries from each treatment group were immunolabeled with Vg-FITC, hybridized with the *Sulcia*-cy5 or *Nasuia*-cy3 probe, and then were observed with a Leica TCS SP5 confocal microscope. Thirty ovaries were observed with a Leica M165C stereomicroscope to calculate the number of ovarioles at different developmental stages. The accumulation levels of NcVgR, NcVg, *Sulcia* OMP, and *Nasuia* porin in 30 insects were detected by RT-qPCR and Western blot assays after treatment of dsNcVgR or dsgfp, respectively. Relative levels of gene expression were normalized to a housekeeping gene *elongation factor 1alpha* gene (EF1, GenBank accession no. AB836665) and estimated by the 2^−△△^*^Ct^* (cycle threshold).

After dsRNA microinjection, 30 adult female leafhoppers were picked out for mating one to one with male leafhoppers in glass tubes containing one rice seedling. The total number of eggs laid by each mated female were recorded to evaluate the female fecundity. The egg hatching rates were evaluated according to the number of neonates/total number of eggs. The longevity of females was also measured. The entire experiment was repeated three times.

### Knocking down the *in vivo* expression of Ncprp in *N. cincticeps*.

We cloned the full-length Ncprp cDNA based on the genomic database of *Nasuia*. The role of *Nasuia* abundance in the oocyte entry process of NcVg was examined by knocking down expression of the Ncprp gene. The abdomens of newly emerged adult females were microinjected with dsRNAs targeting the Ncprp gene (dsNcprp) or dsgfp. The ovaries dissected from 50 insects 6 days after microinjection were immunolabeled with NcVg-FITC, hybridized with the *Sulcia*-cy5 and *Nasuia*-cy3 probes, and then were viewed by confocal microscopy. The proportion of *Nasuia* in the epithelial plugs of the ovaries was calculated depending on fluorescence signal. The levels of Ncprp, *Nasuia*, and *Sulcia* in 30 insect ovaries were measured by RT-qPCR assay (Table S3). The relative levels of gene expression were normalized to the level of the EF1 gene and estimated by the 2^−△△^*^Ct^* (cycle threshold) method. The protein expression levels of Ncprp, *Nasuia*, and *Sulcia* in 30 insect ovaries were detected using a Western blot assay. In addition, the mortality, fecundity, and egg hatching rates of *N. cincticeps* treated with dsgfp or dsNcprp were monitored following the same protocol for dsNcVgR treatment. The experiments were repeated three times.

### Data analyses.

All data were analyzed with SPSS (version 17.0; SPSS, USA). Percentage data were first transformed by the arcsine square root before analysis. Data sets were examined for normality before comparison. Multiple comparisons of the means were conducted using a one-way analysis of variance (ANOVA) followed by Tukey’s honestly significant difference (HSD) test at the *P* < 0.05 significance level. Comparisons between two means were conducted using an independent *t* test.

10.1128/mBio.01142-20.10 TEXT S1 Supplemental Materials and Methods. Detailed description of insect and antibody preparation, Western blot assay, absolute RT-qPCR, preparation of dsRNA, and phylogenetic trees. Download Text S1, DOCX file, 0.03 MB. Copyright © 2020 Mao et al. 2020 Mao et al. This content is distributed under the terms of the Creative Commons Attribution 4.0 International license.

### Data availability.

Sequence data were deposited in GenBank under accession no. MK722101.
