# Supplementary material for: Insect Bacterial Symbiont-Mediated Vitellogenin Uptake into Oocytes To Support Egg Development
Source: mBio. 2020 Nov 10;11(6):e01142-20. doi: 10.1128/mBio.01142-20 (PMC7667026; doi:10.1128/mBio.01142-20)
Supplement: TABLE S1 [file mBio.01142-20-st001.docx]

**Table S1.** Localization of NcVg, *Sulcia* and *Nasuia* in hemocytes of female *N. cincticeps*, as revealed by cofocal microscopy.

| Insect no. | Total hemocytes | *Sulcia* signals^*^ | *Nasuia* signals^*^ | NcVg signals^*^ |
| --- | --- | --- | --- | --- |
| 1 | 35 | 0 | 24 | 35 |
| 2 | 31 | 0 | 22 | 31 |
| 3 | 30 | 0 | 25 | 30 |
| 4 | 34 | 0 | 21 | 34 |
| 5 | 28 | 0 | 20 | 28 |
| 6 | 32 | 0 | 25 | 32 |
| 7 | 26 | 0 | 20 | 26 |
| 8 | 25 | 0 | 21 | 25 |
| 9 | 35 | 0 | 25 | 35 |
| 10 | 31 | 0 | 22 | 31 |
| 11 | 31 | 0 | 22 | 31 |
| 12 | 32 | 0 | 25 | 32 |
| 13 | 34 | 0 | 23 | 34 |
| 14 | 29 | 0 | 24 | 29 |
| 15 | 27 | 0 | 22 | 27 |
| 16 | 33 | 0 | 24 | 33 |
| 17 | 28 | 0 | 23 | 28 |
| 18 | 27 | 0 | 25 | 27 |
| 19 | 32 | 0 | 21 | 32 |
| 20 | 32 | 0 | 25 | 32 |
| 21 | 33 | 0 | 22 | 33 |
| 22 | 35 | 0 | 23 | 35 |
| 23 | 25 | 0 | 22 | 25 |
| 24 | 25 | 0 | 23 | 25 |
| 25 | 31 | 0 | 22 | 31 |
| 26 | 34 | 0 | 23 | 34 |
| 27 | 33 | 0 | 24 | 33 |
| 28 | 25 | 0 | 23 | 25 |
| 29 | 34 | 0 | 23 | 34 |
| 30 | 29 | 0 | 21 | 29 |

^*^ One positive fluorescence spot was considered as one signal unit. The leafhopper hemocyte smears were stained with *Sulcia*-cy5, *Nasuia*-cy3 and NcVg-FITC and observed using confocal microscopy under the same optical settings.
