# Supplementary material for: Insect Bacterial Symbiont-Mediated Vitellogenin Uptake into Oocytes To Support Egg Development
Source: mBio. 2020 Nov 10;11(6):e01142-20. doi: 10.1128/mBio.01142-20 (PMC7667026; doi:10.1128/mBio.01142-20)
Supplement: TABLE S2 [file mBio.01142-20-st002.docx]

**Table S2.** Distribution of NcVg in *Sulcia* or *Nasuia* in ovaries of female *N. cincticeps*

|  | NcVg in *Sulcia* or *Nasuia* in ovaries as revealed by confocal microscopy | | | | |  | NcVg in *Sulcia* or *Nasuia* in ovaries as revealed by immunoelectron microscopy | | | | | |
| --- | --- | --- | --- | --- | --- | --- | --- | --- | --- | --- | --- | --- |
|  | *Nasuia* signals | |  | *Sulcia* signals | |  |  | *Nasuia* | |  | *Sulcia* | |
| Insect | Total | NcVg signals^*^ |  | Total | NcVg signals^*^ |  | Insect | Total | NcVg signals^#^ |  | Total | NcVg signals^#^ |
| 1 | 12 | 10 |  | 14 | 0 |  | 1 | 4 | 3 |  | 6 | 0 |
| 2 | 12 | 9 |  | 15 | 0 |  | 2 | 6 | 4 |  | 7 | 0 |
| 3 | 13 | 11 |  | 13 | 0 |  | 3 | 5 | 4 |  | 6 | 0 |
| 4 | 18 | 18 |  | 21 | 0 |  | 4 | 7 | 6 |  | 10 | 0 |
| 5 | 10 | 8 |  | 15 | 0 |  | 5 | 5 | 4 |  | 7 | 0 |
| 6 | 22 | 17 |  | 17 | 0 |  | 6 | 8 | 7 |  | 9 | 0 |
| 7 | 23 | 18 |  | 28 | 0 |  | 7 | 7 | 5 |  | 8 | 0 |
| 8 | 28 | 22 |  | 31 | 0 |  | 8 | 9 | 6 |  | 7 | 0 |
| 9 | 14 | 11 |  | 26 | 0 |  | 9 | 3 | 2 |  | 5 | 0 |
| 10 | 25 | 20 |  | 34 | 0 |  | 10 | 7 | 4 |  | 7 | 0 |
| 11 | 24 | 19 |  | 30 | 0 |  | 11 | 4 | 2 |  | 6 | 0 |
| 12 | 27 | 20 |  | 35 | 0 |  | 12 | 6 | 4 |  | 8 | 0 |
| 13 | 21 | 16 |  | 21 | 0 |  | 13 | 8 | 5 |  | 7 | 0 |
| 14 | 13 | 9 |  | 13 | 0 |  | 14 | 4 | 2 |  | 3 | 0 |
| 15 | 18 | 14 |  | 25 | 0 |  | 15 | 8 | 7 |  | 11 | 0 |
| 16 | 17 | 14 |  | 21 | 0 |  | 16 | 9 | 6 |  | 9 | 0 |
| 17 | 25 | 17 |  | 23 | 0 |  | 17 | 4 | 3 |  | 6 | 0 |
| 18 | 27 | 22 |  | 38 | 0 |  | 18 | 4 | 3 |  | 5 | 0 |
| 19 | 19 | 10 |  | 28 | 0 |  | 19 | 7 | 6 |  | 9 | 0 |
| 20 | 20 | 15 |  | 29 | 0 |  | 20 | 3 | 2 |  | 4 | 0 |
| 21 | 26 | 15 |  | 40 | 0 |  | 21 | 4 | 3 |  | 6 | 0 |
| 22 | 13 | 11 |  | 13 | 0 |  | 22 | 5 | 3 |  | 5 | 0 |
| 23 | 16 | 13 |  | 23 | 0 |  | 23 | 6 | 5 |  | 6 | 0 |
| 24 | 30 | 24 |  | 35 | 0 |  |  |  |  |  |  |  |
| 25 | 16 | 13 |  | 12 | 0 |  |  |  |  |  |  |  |

^*^ One positive fluorescence spot was considered as one signal unit. The leafhopper ovaries were immunofluorescence stained with *Sulcia*-cy5, *Nasuia*-cy3 and NcVg-FITC and observed using confocal microscopy under the same optical settings.

^#^ One bacterial symbiont that labelled by more than 10 gold particles was considered as one signal unit. The ovary samples were ultrathin sectioned and immunolabelled with NcVg-specific IgG as primary antibody, followed by treatment with 15-nm gold particle-conjugated goat antibodies against rabbit IgG as secondary antibody.
