# Supplementary material for: Insect Bacterial Symbiont-Mediated Vitellogenin Uptake into Oocytes To Support Egg Development
Source: mBio. 2020 Nov 10;11(6):e01142-20. doi: 10.1128/mBio.01142-20 (PMC7667026; doi:10.1128/mBio.01142-20)
Supplement: TABLE S3 [file mBio.01142-20-st003.docx]

**Table S3.** Primers used in this study.

| Oligonucleotide | Assay | Sequence (5’-3’) |
| --- | --- | --- |
| EF1_F | qPCR | CAGTGAGAGCCGTTTTGAG |
| EF1_R | qPCR | AGGGCATCTTGTCAGAGGGC |
| *Nasuia*_F | qPCR | GGGGAAAACCTCGCGTTATA |
| *Nasuia*_R | qPCR | CCACTGCTGCCTCTCGTAAG |
| *Sulcia*_F | qPCR | GGGGACTCTAATAAGACTGC |
| *Sulcia*_R | qPCR | CTGAGATCGGCTTTCTGGAT |
| Ncprp_F | qPCR | TACCAACAGAGGACTCCCTT |
| Ncprp_R | qPCR | AGTCGTCGTCTAGCCAGTCG |
| NcVg_F | qPCR | AAGTTTTCAGAGGACAACAGTT |
| NcVg_R | qPCR | TCTTGA GGAGCGGTGAAGTC |
| NcVgR_F | qPCR | GATGGCCTAGTCTGCAATCCAAAT |
| NcVgR_R | qPCR | TCGGTTCAAACCTAAGCTAAAATG |
| *Nasuia*_porin_F | Y2H | GGCCATGGAGGCCATGTCAAATTCAATTTATTT TTTTTTTATAG |
| *Nasuia*_porin_R | Y2H | GGCCTCCATGGCCTTAATCTAATAATTTTAAAAATTTTTTATAATAAG |
| *Sulcia*_OMP_F | Y2H | AGTGAATTCCACATGTATGGAGATAATCAAAAAATTATTTTTA |
| *Sulcia*_OMP_R | Y2H | ATGGATCCCGTACTAATATGCAATATCACATCCTAAAAAT |
| NcVg1_F | Y2H | GGCCATGGAGGCCAAATCCAACACCCAATACAAGTAC |
| NcVg1_R | Y2H | GGCCTCCATGGCCATTTACTGAGGAGAGCCACTGGCAG |
| NcVg2_F | Y2H | GGCCATGGAGGCCTTGGTTTATGAGTACAATCCGGC |
| NcVg2_R | Y2H | GGCCTCCATGGCCATTTATTTGATGGCAGACTGGACAGC |
| NcVg3_F | Y2H | GGCCATGGAGGCCTTCTCCAAGCGTCACATGCGT |
| NcVg3_R | Y2H | GGCCTCCATGGCCATTTAGGAGGTGTACGGCTCGGTG |
| NcVg4_F | Y2H | GGCCATGGAGGCCGAAGTTTTCAGAGGACAACAGTT |
| NcVg4_R | Y2H | GGCCTCCATGGCCATTTAGTCTTGAGGAGCGGTGAAGTC |
| NcVgR_F | RNAi | GGATCCTAATACGACTCACTATAGGGAAGCGAGCTGACATGTCTG |
| NcVgR_R | RNAi | GGATCCTAATACGACTCACTATAGGTGGTTGCATTGATTGTGGCA |
| Ncprp_F | RNAi | GGATCCTAATACGACTCACTATAGGATGCGTTATCATAAGGACTTTG |
| Ncprp_R | RNAi | GGATCCTAATACGACTCACTATAGGTTGTGGCTGAGTTGGCGGGG |
| gfp_F | RNAi | GGATCCTAATACGACTCACTATAGGGAAGTTCACCTTGATGCCGTT |
| gfp_R | RNAi | GGATCCTAATACGACTCACTATAGGAAGTTCAGCGTGTCCGGCGA |
| *Nasuia*_porin_F | Pull down | CGTGGATCCCCATGTCAAATTCAATTTATTT TTTTTTTATAG |
| *Nasuia*_porin_R | Pull down | CGATGAATTCCCTTAATCTAATAATTTTAAAAATTTTTTATAATAAG |
| *Sulcia*_OMP_F | Pull down | CGTGGATCCCCATGTATGGAGATAATCAAAAAATTATTTTTA |
| *Sulcia*_OMP_R | Pull down | CGATGAATTCCCCTAATATGCAATATCACATCCTAAAAAT |
| NcVg2_F | Pull down | GGGGACAAGTTTGTACAAAAAAGCAGGCTTCTTGGTTTATGAGTACAATCCGGC |
| NcVg2_R | Pull down | GGGGACCACTTTGTACAAGAAAGCTGGGTCTTTGATGGCAGACTGGACAGC |
