# Supplementary material for: Insect Bacterial Symbiont-Mediated Vitellogenin Uptake into Oocytes To Support Egg Development
Source: mBio. 2020 Nov 10;11(6):e01142-20. doi: 10.1128/mBio.01142-20 (PMC7667026; doi:10.1128/mBio.01142-20)
Supplement: TEXT S1 [file mBio.01142-20-s0001.docx]

**SUPPLEMENTAL MATERIALS AND METHODS**

**Insect and antibody preparation**

The leafhopper *N. cincticeps* was originally collected from rice fields in Fujian, China. The leafhopper *R. dorsalis*, *N. nigropictus* and *N. virescens* were collected from Guangdong, China. The four leafhopper species were maintained in growth chamber at 28 ± 1°C under a 16: 8 h light: dark photoperiod and 60% relative humidity on TN-1 rice plants.

Rabbit polyclonal antibody against NcVg was prepared. Briefly, the gene sequences of the three domains of NcVg were synthesized and cloned into the prokaryotic expression vector pDEST17. The recombinant plasmids were transformed to *E*sch*e*richia *coli* strain Rosetta to express the fusion proteins. Subsequently, the fusion proteins were injected into rabbit to produce antibody. Rabbit polyclonal antibody against NcVgR and mouse polyclonal antibody against Ncprp were also generated following the same protocol. Mouse polyclonal antibodies against *Nasuia* porin and *Sulcia* OMP had been prepared previously (1, 2). IgGs were isolated from specific polyclonal antisera using a protein A-Sepharose affinity column (Thermofisher). Purified IgGs were directly conjugated to fluorescein isothiocyanate (FITC) or rhodamine according to the manufacturer’s instructions (Invitrogen).

**Western blot assay**

Protein samples were subjected to SDS-PAGE and transferred onto PVDF membranes (Millipore, USA). The membrane was blocked for 12 h in 5% nonfat dry milk (20 mM Tris-HCl, 500 mM NaCl, and 0.1% Tween 20, pH 7.4) at 4°C, and then was incubated with antibodies against NcVg, *Nasuia* porin or *Sulcia* OMP (1:2000) at 4°C overnight. After washing with TBST buffer, the membranes were incubated with HRP-labeled goat anti-rabbit or anti-mouse IgG (H + L) (Invitrogen, 1:10000) at room temperature for 2 h. Positive immunoreaction was visualized using Clarity ECL Blotting Substrate (Bio-Rad) for HRP-based chemiluminescent detection. Images were captured with AI600 (GE Healthcare Life Sciences) and analyzed with ImageQuantTL software (GE Healthcare Life Sciences).

**Absolute RT-qPCR**

Total RNAs were extracted using Trizol reagent (Thermo Fisher), and reverse transcription was performed using a Maxima First Strand cDNA Synthesis Kit (Thermo Fisher). Absolute RT-qPCR was performed with the 2×RealStar Green Fast Mixture (GenStar) following the manufacturer’s instructions in a QuantStudio 5 Real-Time PCR Systems (Thermo Fisher). Cycle thresholds (CTs) were obtained by the absolute RT-qPCR assay. The NcVg transcript level and 16S rRNA gene copy number of *Nasuia* and *Sulcia* were calculated as the log of the number of copies per microgram of total RNAs by mapping the CT value to the standard curve of the NcVg gene (y=-3.5764x+35.720), *Sulcia* 16S rRNA sequence (y=-3.5839x+38.096) and *Nasuia* 16S rRNA sequence (y=-3.2172x+40.029), respectively. The significance of comparisons of NcVg transcript levels and 16S rRNA gene copy number of *Nasuia* and *Sulcia* in insects at different days post emergence was analyzed by one-way analysis of variance with Tukey’s HSD test with Graphpad prism 7.0 software (Graphpad Prism, Graphpad Prism Software Inc., San Diego, USA).

**Preparation of dsRNA**

To perform RNA interference (RNAi) experiments, the dsRNAs targeting NcVgR gene (dsNcVgR), gfp gene (dsgfp) and Ncprp gene (dsNcprp) were synthesized. Primers were designed according to the sequences of *N. cincticeps* NcVgR (GenBank accession no. KX022098.1), gfp (GenBank accession no. U55761.1) and proline-rich protein (Ncprp) (GenBank accession no. MK722101). The primer sequences were fused with the T7 promoter sequence, and DNA fragments with the T7 promoter sequence on both sides were amplified by PCR (Table S3). The PCR products were used to synthesize dsRNA with Promega RiboMax™ T7 system (Promega) following the protocol. After phenol-chloroform extraction and heat treatment, the dsRNAs were diluted with nuclease-free water for microinjection.

**Phylogenetic Trees**

Evolutionary relationships of 16S RNA sequences of *Nasuia* of different leafhopper species were constructed using MEGA7 (3). The bootstrapped (1000 replicates) trees were inferred using the neighbor-joining (NJ) method with evolutionary distances computed using the Kimura 2-parameter method. All positions containing gaps and missing data were eliminated.

**References**

1. Jia D, Mao Q, Chen Y, Liu Y, Chen Q, Wu W, Zhang X, Chen H, Li Y, Wei T. 2017. Insect symbiotic bacteria harbour viral pathogens for transovarial transmission. Nat Microbiol 2:17025.
2. Wu W, Huang L, Mao Q, Wei J, Li J, Zhao Y, Zhang Q, Jia D, Wei T. 2019. Interaction of viral pathogen with porin channels on the outer membrane of insect bacterial symbionts mediates their joint transovarial transmission. Philos Trans R Soc Lond B Biol Sci 374:20180320.
3. Kumar S, Stecher G, Tamura K. 2016. MEGA7: Molecular Evolutionary Genetics Analysis Version 7.0 for Bigger Datasets. Mol Biol Evol, 7:1870.
